# Multivariate curve resolution of time course microarray data

**DOI:** 10.1186/1471-2105-7-343

**Published:** 2006-07-13

**Authors:** Peter D Wentzell, Tobias K Karakach, Sushmita Roy, M Juanita Martinez, Christopher P Allen, Margaret Werner-Washburne

**Affiliations:** 1 Department of Chemistry, Dalhousie University, Halifax, NS B3H 4J3, Canada; 2 Department of Biology, University of New Mexico, Albuquerque, NM 87131, USA

## Abstract

**Background:**

Modeling of gene expression data from time course experiments often involves the use of linear models such as those obtained from principal component analysis (PCA), independent component analysis (ICA), or other methods. Such methods do not generally yield factors with a clear biological interpretation. Moreover, implicit assumptions about the measurement errors often limit the application of these methods to log-transformed data, destroying linear structure in the untransformed expression data.

**Results:**

In this work, a method for the linear decomposition of gene expression data by multivariate curve resolution (MCR) is introduced. The MCR method is based on an alternating least-squares (ALS) algorithm implemented with a weighted least squares approach. The new method, MCR-WALS, extracts a small number of basis functions from untransformed microarray data using only non-negativity constraints. Measurement error information can be incorporated into the modeling process and missing data can be imputed. The utility of the method is demonstrated through its application to yeast cell cycle data.

**Conclusion:**

Profiles extracted by MCR-WALS exhibit a strong correlation with cell cycle-associated genes, but also suggest new insights into the regulation of those genes. The unique features of the MCR-WALS algorithm are its freedom from assumptions about the underlying linear model other than the non-negativity of gene expression, its ability to analyze non-log-transformed data, and its use of measurement error information to obtain a weighted model and accommodate missing measurements.

## Background

In recent years there has been an increased interest in the study of serial microarray experiments, particularly time course data. This has been driven by the greater availability of such data and the appeal of elucidating the temporal relationships among genes. Often, approaches to the analysis of these data sets have employed traditional methods of exploratory data analysis and clustering, but it has been recognized that methods specifically designed to exploit the temporal relationships are advantageous [1]. This has led to approaches based on time series and frequency analysis, hidden Markov models, and linear modeling, among others.

One popular strategy in modeling time course data will be referred to here as bilinear modeling. In this approach, the matrix of gene expression data, **X **(*m *genes × *n *experiments), is represented as the product of two lower rank matrices, which we will designate as **C **and **P**, and a residual error term, **E**:

**X **= **CP **+ **E **    (1)

(Note: In this work, matrices will be represented by bold upper-case fonts, vectors by bold lower case fonts, and scalars by italics.) In this representation, **C **has dimensions *m *× *p *and **P **has dimensions *p *× *n*, where *p *is the number of basis vectors (also referred to as factors or components) needed to reconstruct the data within experimental uncertainty. Normally, for microarray experiments, the number of genes (*m*) is much greater than the number of experiments (*n*), which in turn is greater than *p*. In general, the first goal in bilinear modeling is to obtain the matrices **C **and **P**, given the experimental data, **X**, and some knowledge or assumptions of the statistical characteristics of **E**. However, for a given data set there are an infinite number of degenerate solutions for **C **and **P **due to arbitrary rotation (rotational ambiguity) and scaling (scale ambiguity) of the basis vectors in the subspace they define. To overcome this problem, some of the approaches commonly adopted in the microarray literature include expression deconvolution, principal components analysis (PCA) and independent components analysis (ICA), among others.

In expression deconvolution [2], the rotational ambiguity problem in Eq. (1) is addressed by assuming that the matrix **C **is already known. Typically, the vectors that constitute the columns of **C **would be estimated from cells known to be associated with a specific cellular state, such as certain phases of the cell cycle. Once **C **is known, the solution for **P **becomes a classical least squares problem. Although this approach is quite straightforward, its major drawback is that it requires complete knowledge of one of the constituent matrices, information which is not always available. This information is not required for PCA [3-6], which uses singular value decomposition (SVD) to decompose the expression matrix into a set of scores (**C**) and loadings (**P**) that are truncated to the first *p *factors. PCA imposes the constraint that successive factors in the decomposition must (a) account for the largest amount of residual variance, and (b) be orthogonal to all of the factors determined to that point. Because of these constraints, the scores and loadings vectors do not normally have an obvious biological interpretation. Despite this shortcoming, the extension of SVD to compare expression profiles across different data sets has been reported [7,8]. The strategy employed by ICA [9-11] in the decomposition of **X **is similar to that used by PCA, except that the constraints require a minimization of the statistical dependence of the columns of **C**. Although one might expect this constraint to produce more meaningful factors than PCA, the biological rationale behind its imposition has not been clearly established. Other bilinear modeling approaches have also been used (*e.g*. [12,13]), but these will not be described in detail, except where they relate to the current work below.

In this work, an alternative approach to solving the bilinear modeling problem represented by Equation (1) is described and evaluated. This problem is not new and arises in many disciplines, leading to a variety of solutions. In chemistry, the problem often presents itself in the analysis of chemical mixtures, where neither the concentration nor the identity of the constituents is known. Solutions to this problem, collectively referred to as multivariate curve resolution (MCR) methods [14,15], impose constraints on the results that are physically meaningful. The simplest and most common of these is a requirement of non-negativity in the elements of **C **and **P**. Other constraints include unimodality, equality, and closure. Quite often, the imposition of one or more constraints is sufficient to produce a unique or nearly unique solution to the rotational ambiguity problem. (It should be noted that, in the absence of additional information, it is impossible to resolve the scale ambiguity, so an arbitrary normalization is normally applied to the basis vectors in either **C **or **P**.) We show that this approach, with some modifications necessitated by the nature of microarray data, can be successfully adapted to study gene expression.

### A simple biological model

**Figure 1:**
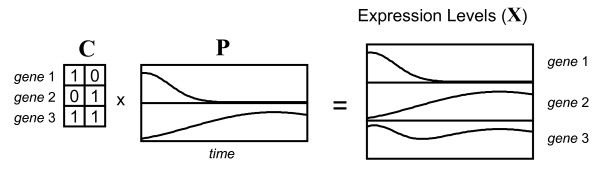
**Simplified representation of the bilinear model**. The expression levels of three genes as a function of time are represented as the linear combination of two underlying regualtory factors making up the profile matrix, **P**, and the contribution matrix, **C**, which determines how each gene responds to the individual regulatory factors.

To rationalize the bilinear model in Equation (1) from a biological perspective, Figure 1 shows a simple framework illustrated for the case of only three genes and two underlying factors or components. We will refer to the matrix **P **generically as the "profile matrix" and it can be viewed as representing the evolution of regulatory inputs (transcription factors, promoters, promoter/suppressor combinations) as a function of time. In other work, analogous terms have been used to describe the vectors of the profile matrix: "process objects" [12]; "transcription module" [13]; "biological processes" [10]; "arraylets" [7]; "eigenarrays" [5]. In all of these instances, a fundamental assumption is that "the coregulation of genes may be described by a small number of effective regulators, each acting on a large set of genes and varying between distinct biological situations" [9]. The matrix **C **will be referred to as the "contribution matrix" and describes how each gene responds to each of the regulating factors. In a conceptual interpretation, these could correspond to receptor elements on a particular gene. In the example given in Figure 1, gene 1 responds only to the first transcription factor and gene 2 only to the second, while gene 3 responds in equal measure to both regulators. Although only values of unity or zero are used in the example for simplicity, the model in its general form does not impose this restriction. The elements of **C **are analogously referred to in a variety of ways in other work: "gene objects" [12]; "independent components" [9]; "pure populations" [2]; "genelets" [7]; "eigengenes" [5]. The expression profile for each gene is therefore represented as a linear combination of the vectors in the profile matrix in proportions defined by the gene's contribution values. If one could extract the profile matrix from the expression data, it would provide important information regarding the underlying regulatory inputs driving gene expression. Likewise, a knowledge of the contribution matrix would illuminate relationships among the genes in the organism.

The model represented in Figure 1 is amenable to solution through the implementation of non-negativity constraints for the elements of **C **and **P**, since a regulatory input can turn the expression of a gene on or off, but it cannot result in negative expression. Therefore, it should be possible to apply MCR with non-negativity constraints to expression data, with one important caveat – it cannot be directly applied to log-transformed data. The implications of this are discussed in the next section.

### Implications of data transformation

While the level of gene expression cannot physically be negative, this is not true of the logarithms, so non-negativity constraints in MCR cannot be applied to log-transformed data. While this represents one limitation of log-transformed data, there is another implication of imposing the transformation that is perhaps more important. It should be apparent that the simple linear relationship represented in Figure 1, where the level of expression of a gene is presumed to be in direct proportion to the abundance of contributing regulatory factors, would no longer be valid under logarithmic transformation. Despite this fact, there appears to be little discussion in the literature regarding the actual representation of expression values. We have found that many of the authors studying applications of bilinear modeling methods do not explicitly state whether log-transformed values were used, but those who did generally used transformed data, implying that this is the norm. There is a limited discussion of the effects of the log transformation on the linear model [10,11]. These authors point out that a linear model in the log-transformed domain corresponds to a multiplicative model in the untransformed domain; *i.e*. the expression of a gene is in proportion to the product of two or more regulating factors. While such cooperative effects are entirely possible and even likely, the simple linear model represented by Figure 1 seems to us to be a more intuitive construct for a first approximation. Lee *et al*. [10] suggested the use of nonlinear mapping to resolve this problem. Kreil *et al*. [11] compared the results of applying ICA to transformed and untransformed data and found lower reconstruction errors in the log-transformed space. They suggest that a possible reason for this was the structure of measurement errors in the two spaces.

One of the reasons for the popularity of log-ratio as opposed to ratio data in representing gene expression is the error structure of raw expression data, which is generally accepted to have a multiplicative component (see for example [16]). Because uncertainty in the intensity ratio is typically proportional to the magnitude of the value, log-transformation gives rise to values with a uniform error variance. Moreover, transformation reduces the influence of outliers, which are common in microarray experiments. Because most bilinear modeling algorithms are based on least-squares minimization, the effects of heteroscedastic measurement error and outliers can be large. Derived models will tend to emphasize large measurements, even though smaller measurements may contain an equivalent amount of information. This problem can be exacerbated with time-course experiments, where variations in expression can be genome-wide and the reference *m*RNA does not always bear a close expression match to the test *m*RNA.

Based on the view that the linear model presented in Figure 1 seems more natural from a biological perspective, and our desire to impose non-negativity constraints, modeling in this work was conducted on untransformed ratios. It was clear that, in order to do this, some method would be need to be developed to accommodate the multiplicative error structure and outliers in the data. Liu *et al*. [6] employed a robust form of SVD to address the problem of outliers, but avoided the multiplicative noise issue by applying it to log-transformed data. Except in special circumstances, the problem of non-uniform measurement noise cannot be addressed through simple scaling. However, in recent years, a number of techniques, such as maximum likelihood PCA (MLPCA) [17] and total least squares (TLS) [18], have been developed to treat heteroscedastic and correlated error structures. In this work, we have adapted a TLS approach to MCR and demonstrate its performance through its application to widely studied yeast cell cycle data.

### Multivariate curve resolution

MCR attempts to solve Eq. (1) for its two constituent matrices based on a prior knowledge of the number of underlying factors, *p*, and any constraints on the system. Generally, non-negativity constraints are assumed and additional constraints are added as required by the problem. In early work, Lawton and Sylvestre [19] developed an analytical solution for boundaries of solution vectors in the case of two factors, but direct solution for more than two components is made impractical due to the complexities of the problem. A wide range of alternative strategies have been developed since that time, but one of the most popular approaches due to its simplicity and reliability is alternating least squares (ALS) [20]. This is the approach used in this work.

The basic algorithm for curve resolution by ALS is as follows. Initially, one must choose the number of factors (components), *p*, that will be extracted for the bilinear model. A variety of approaches can be used for this, many of which are based on the statistics of reconstructing the original data from PCA scores and loadings with increasing numbers of factors [21]. Alternatively, one can examine the results of curve resolution applied with different numbers of factors, seeking results that show a pattern of behavior consistent with the system under study. For example, in a time course study, one would expect that profiles extracted will show a smoothly varying function (assuming sufficient sampling). The appearance of random patterns would suggest that one has reached the point where noise is being modeled.

Once the number of factors has been chosen, an initial estimate for **C **or **P **must be provided. Because of the symmetry of the algorithm, either of the matrices can be the starting point, but in this application **P **is suggested since it will be smaller and follow a more systematic variation. One disadvantage of the ALS algorithm is that the selection of this initial matrix can influence the final solution, in part because this solution may represent only one of a range of feasible solutions. The variability of these solutions will depend somewhat on the structure of the data, but in many cases they will fall into fairly tight boundaries. There are several approaches to defining the initial **P**. One is to simply assign random positive values to the elements. While this ensures the results will be unbiased, the initial vectors are almost certainly well outside the subspace of the measurements and therefore convergence may be slower and more prone to numerical difficulties. Another fairly simple approach is to use *p *profiles selected randomly from **X**. These will be close to the true subspace, but may have some problems with collinearity. Other more systematic methods, such as SIMPLISMA [22] and the needle-search method [23], can also be used. Whichever method is used, it is advisable to run the algorithm several times from different starting points to ensure consistency in the generated profiles.

At each stage in the algorithm where a new estimate of **P **is generated, it is scaled so that the Euclidean norm of each row is equal to unity. This is necessary because of the scale ambiguity that results from the fact that the columns of **C **and the rows of **P **can be arbitrarily scaled relative to each other to give the same result for **X**. Because of this, the absolute magnitudes of the rows of **P **and the columns of **C **are not meaningful except in a relative sense within each vector. This ambiguity can only be resolved if separate absolute standards are available, but generally the relative magnitudes are more important in any case. To avoid infinite degenerate solutions that differ only by a scaling factor, the ambiguity requires that one of the matrices be scaled to a fixed point of reference so that convergence can be determined. In this case, the profile vectors are scaled to unit length, but other criteria, such as unit area, could also have been used.

The iterative part of the ALS algorithm begins when an estimate of **C **is calculated based on the initial estimate of **P **and the microarray data in **X**. This can be obtained in the usual way, solving the least-squares problem using the pseudo-inverse of the estimated **P**,

C^=XP^T(P^P^T)−1     (2)
 MathType@MTEF@5@5@+=feaafiart1ev1aaatCvAUfKttLearuWrP9MDH5MBPbIqV92AaeXatLxBI9gBaebbnrfifHhDYfgasaacH8akY=wiFfYdH8Gipec8Eeeu0xXdbba9frFj0=OqFfea0dXdd9vqai=hGuQ8kuc9pgc9s8qqaq=dirpe0xb9q8qiLsFr0=vr0=vr0dc8meaabaqaciaacaGaaeqabaqabeGadaaakeaaieqacuWFdbWqgaqcaiabg2da9iab=Hfayjqb=bfaqzaajaWaaWbaaSqabeaacqqGubavaaGccqGGOaakcuWFqbaugaqcaiqb=bfaqzaajaWaaWbaaSqabeaacqqGubavaaGccqGGPaqkdaahaaWcbeqaaiabgkHiTiabigdaXaaakiaaxMaacaWLjaWaaeWaceaacqaIYaGmaiaawIcacaGLPaaaaaa@3DFE@

In order to observe non-negativity constraints, the negative values in C^
 MathType@MTEF@5@5@+=feaafiart1ev1aaatCvAUfKttLearuWrP9MDH5MBPbIqV92AaeXatLxBI9gBaebbnrfifHhDYfgasaacH8akY=wiFfYdH8Gipec8Eeeu0xXdbba9frFj0=OqFfea0dXdd9vqai=hGuQ8kuc9pgc9s8qqaq=dirpe0xb9q8qiLsFr0=vr0=vr0dc8meaabaqaciaacaGaaeqabaqabeGadaaakeaaieqacuWFdbWqgaqcaaaa@2DD1@ are set to zero once this result is obtained. Alternatively, a more rigorous solution to the non-negative least squares (NNLS) problem can be obtained using standard methods [24] which minimize the sum of squares of residuals in **X **conditional on the constraint that the elements in **C **are greater than or equal to zero. Following this step, the estimated **C **matrix is used to re-estimate **P**. Once again, this can be done by censoring the standard least squares solution,

P^=(C^TC^)−1C^TX     (3)
 MathType@MTEF@5@5@+=feaafiart1ev1aaatCvAUfKttLearuWrP9MDH5MBPbIqV92AaeXatLxBI9gBaebbnrfifHhDYfgasaacH8akY=wiFfYdH8Gipec8Eeeu0xXdbba9frFj0=OqFfea0dXdd9vqai=hGuQ8kuc9pgc9s8qqaq=dirpe0xb9q8qiLsFr0=vr0=vr0dc8meaabaqaciaacaGaaeqabaqabeGadaaakeaaieqacuWFqbaugaqcaiabg2da9iabcIcaOiqb=neadzaajaWaaWbaaSqabeaacqqGubavaaGccuWFdbWqgaqcaiabcMcaPmaaCaaaleqabaGaeyOeI0IaeGymaedaaOGaf83qamKbaKaadaahaaWcbeqaaiabbsfaubaakiab=HfayjaaxMaacaWLjaWaaeWaceaacqaIZaWmaiaawIcacaGLPaaaaaa@3DCC@

or by solving the NNLS problem. The rows of P^
 MathType@MTEF@5@5@+=feaafiart1ev1aaatCvAUfKttLearuWrP9MDH5MBPbIqV92AaeXatLxBI9gBaebbnrfifHhDYfgasaacH8akY=wiFfYdH8Gipec8Eeeu0xXdbba9frFj0=OqFfea0dXdd9vqai=hGuQ8kuc9pgc9s8qqaq=dirpe0xb9q8qiLsFr0=vr0=vr0dc8meaabaqaciaacaGaaeqabaqabeGadaaakeaaieqacuWFqbaugaqcaaaa@2DEB@ are normalized as described above following this step, and the procedure is repeated, estimating **C **once again from **P**. Eqs. (2) and (3) represent the core of the ALS algorithm and give rise to its name, since each step alternately estimates one matrix given the other. The iterations continue until convergence, which is most easily tested by checking for insignificant changes in **P **and/or **C**.

### Weighted multivariate curve resolution

Although the ALS method for multivariate curve resolution works well in many cases, one of the assumptions that it makes in solving the least squares problem is that the residual measurement errors exhibit uniform measurement variance. While this is true, or nearly true, for many spectroscopic methods used in chemistry, the same cannot be said for microarray data. It has been widely observed that microarray intensity measurements, at least for relatively high intensities, exhibit a multiplicative error structure; *i.e*. a constant coefficient of variation [16,25-29]. Through propagation of error, it is easily shown that this proportional error structure in the intensities leads to multiplicative errors in the expression ratios as well. This is problematic for MCR-ALS, since it will tend to ignore the smaller signals, even though they have the same signal-to-noise ratio as the larger signals. This problem is normally addressed through a log transformation, but as noted earlier, this would destroy the bilinear structure of the original expression data and remove the non-negativity constraint used by MCR.

A more general model for the error structure in microarray intensity measurements involves both multiplicative and additive terms [16,27], with the additive term becoming most important for low intensity measurements. As expected, this additive term leads to a very large coefficient of variation in expression ratios for low intensity signals, which do not follow the general multiplicative error structure. Often, these measurements, which are close to the background, are excluded from the analysis, as are spots that are judged to be unacceptable due to their morphology or other reasons. Such missing data can be treated in a number of ways. One approach is to simply eliminate the corresponding gene from all experiments, but this may remove important information if the measurement is unreliable in only one or a few experiments. Therefore, a number of methods have been developed to accommodate missing data through imputation [6,30]. This would be a desirable feature of a curve resolution algorithm as well.

What is needed is a MCR method that is capable of incorporating measurement error information into the data analysis to obtain an optimal solution under these circumstances, in effect weighting each measurement in proportion to its estimated reliability. With such a method, the proportional error structure of microarray measurements could be accommodated so that a ratio change of 0.2 to 0.4 would be given as much weight as a change from 5 to 10. Moreover, missing measurements could be assigned large uncertainties so they would carry no weight in defining the final model and would effectively be imputed from the other data. In addition to the error models assumed here, such an approach should be able to handle any arbitrary error structure presented in microarray data.

The requirements above describe an errors-in-variables problem for linear regression and this problem has been solved in a variety of ways for different fields of study. Perhaps the most general of these is the technique of total least squares (TLS), which provides an optimal solution for **X **in the linear regression problem **Y **= **AX **given that **Y **and **A **can have arbitrary error structures [18]. Typically, the solution proceeds by augmenting **Y **with **A **column-wise and finding the optimal subspace representation of [**Y A**] using maximum likelihood approaches. The reconstructed **Y **and **A **matrices are then used to solve the least squares problem in the usual way. This approach is closely related to the technique of maximum likelihood principal components analysis (MLPCA) which has recently been described in the literature [17,31]. The TLS approach can be incorporated into the existing MCR-ALS curve resolution method by employing a TLS solution in place of the standard least squares solution. The resulting algorithm will be referred to as weighted alternating least squares (MCR-WALS). The algorithm is given in the "Methods" section, so only a brief description is presented here.

To describe how the WALS algorithm works, we will consider the first half of the alternating estimation procedure. In this case, we are given **X**, which has an arbitrary error structure, and P^
 MathType@MTEF@5@5@+=feaafiart1ev1aaatCvAUfKttLearuWrP9MDH5MBPbIqV92AaeXatLxBI9gBaebbnrfifHhDYfgasaacH8akY=wiFfYdH8Gipec8Eeeu0xXdbba9frFj0=OqFfea0dXdd9vqai=hGuQ8kuc9pgc9s8qqaq=dirpe0xb9q8qiLsFr0=vr0=vr0dc8meaabaqaciaacaGaaeqabaqabeGadaaakeaaieqacuWFqbaugaqcaaaa@2DEB@, which is assumed to be known with certainty, and must solve for C^
 MathType@MTEF@5@5@+=feaafiart1ev1aaatCvAUfKttLearuWrP9MDH5MBPbIqV92AaeXatLxBI9gBaebbnrfifHhDYfgasaacH8akY=wiFfYdH8Gipec8Eeeu0xXdbba9frFj0=OqFfea0dXdd9vqai=hGuQ8kuc9pgc9s8qqaq=dirpe0xb9q8qiLsFr0=vr0=vr0dc8meaabaqaciaacaGaaeqabaqabeGadaaakeaaieqacuWFdbWqgaqcaaaa@2DD1@. The TLS solution to the regression problem **X **= C^
 MathType@MTEF@5@5@+=feaafiart1ev1aaatCvAUfKttLearuWrP9MDH5MBPbIqV92AaeXatLxBI9gBaebbnrfifHhDYfgasaacH8akY=wiFfYdH8Gipec8Eeeu0xXdbba9frFj0=OqFfea0dXdd9vqai=hGuQ8kuc9pgc9s8qqaq=dirpe0xb9q8qiLsFr0=vr0=vr0dc8meaabaqaciaacaGaaeqabaqabeGadaaakeaaieqacuWFdbWqgaqcaaaa@2DD1@P^
 MathType@MTEF@5@5@+=feaafiart1ev1aaatCvAUfKttLearuWrP9MDH5MBPbIqV92AaeXatLxBI9gBaebbnrfifHhDYfgasaacH8akY=wiFfYdH8Gipec8Eeeu0xXdbba9frFj0=OqFfea0dXdd9vqai=hGuQ8kuc9pgc9s8qqaq=dirpe0xb9q8qiLsFr0=vr0=vr0dc8meaabaqaciaacaGaaeqabaqabeGadaaakeaaieqacuWFqbaugaqcaaaa@2DEB@ is solved (conceptually) by first augmenting **X **with P^
 MathType@MTEF@5@5@+=feaafiart1ev1aaatCvAUfKttLearuWrP9MDH5MBPbIqV92AaeXatLxBI9gBaebbnrfifHhDYfgasaacH8akY=wiFfYdH8Gipec8Eeeu0xXdbba9frFj0=OqFfea0dXdd9vqai=hGuQ8kuc9pgc9s8qqaq=dirpe0xb9q8qiLsFr0=vr0=vr0dc8meaabaqaciaacaGaaeqabaqabeGadaaakeaaieqacuWFqbaugaqcaaaa@2DEB@ row-wise and finding the optimal *p*-dimensional subspace of the augmented matrix. In this case, it is clear that this subspace is defined by the *p *rows of P^
 MathType@MTEF@5@5@+=feaafiart1ev1aaatCvAUfKttLearuWrP9MDH5MBPbIqV92AaeXatLxBI9gBaebbnrfifHhDYfgasaacH8akY=wiFfYdH8Gipec8Eeeu0xXdbba9frFj0=OqFfea0dXdd9vqai=hGuQ8kuc9pgc9s8qqaq=dirpe0xb9q8qiLsFr0=vr0=vr0dc8meaabaqaciaacaGaaeqabaqabeGadaaakeaaieqacuWFqbaugaqcaaaa@2DEB@, since it is assumed to be known exactly. It remains to find the optimal representation of **X **in this subspace. We will assume that the errors in **X **follow a normal distribution and their variances are described by a companion matrix, **S**, of equal dimensions. The estimation of **X **in the subspace of P^
 MathType@MTEF@5@5@+=feaafiart1ev1aaatCvAUfKttLearuWrP9MDH5MBPbIqV92AaeXatLxBI9gBaebbnrfifHhDYfgasaacH8akY=wiFfYdH8Gipec8Eeeu0xXdbba9frFj0=OqFfea0dXdd9vqai=hGuQ8kuc9pgc9s8qqaq=dirpe0xb9q8qiLsFr0=vr0=vr0dc8meaabaqaciaacaGaaeqabaqabeGadaaakeaaieqacuWFqbaugaqcaaaa@2DEB@ is then given by the maximum likelihood projection of **X**:

x^i·=xi·Σi−1P^T(P^Σi−1P^T)−1P^     (4)
 MathType@MTEF@5@5@+=feaafiart1ev1aaatCvAUfKttLearuWrP9MDH5MBPbIqV92AaeXatLxBI9gBaebbnrfifHhDYfgasaacH8akY=wiFfYdH8Gipec8Eeeu0xXdbba9frFj0=OqFfea0dXdd9vqai=hGuQ8kuc9pgc9s8qqaq=dirpe0xb9q8qiLsFr0=vr0=vr0dc8meaabaqaciaacaGaaeqabaqabeGadaaakeaaieqacuWF4baEgaqcamaaBaaaleaacqWGPbqAcqWIpM+zaeqaaOGaeyypa0Jae8hEaG3aaSbaaSqaaiabdMgaPjabl+y6NbqabaacceGccqGFJoWudaqhaaWcbaGaemyAaKgabaGaeyOeI0IaeGymaedaaOGaf8huaaLbaKaadaahaaWcbeqaaiabbsfaubaakiabcIcaOiqb=bfaqzaajaGae43Odm1aa0baaSqaaiabdMgaPbqaaiabgkHiTiabigdaXaaakiqb=bfaqzaajaWaaWbaaSqabeaacqqGubavaaGccqGGPaqkdaahaaWcbeqaaiabgkHiTiabigdaXaaakiqb=bfaqzaajaGaaCzcaiaaxMaadaqadiqaaiabisda0aGaayjkaiaawMcaaaaa@51C6@

In this equation, **x**_*i*• _indicates the *i*th row of **X **and **Σ**_*i *_is the corresponding error covariance matrix, given by a diagonal matrix whose diagonal elements are the *i*th row of **S**. The maximum likelihood projection weights the projection of each row of **X **into P^
 MathType@MTEF@5@5@+=feaafiart1ev1aaatCvAUfKttLearuWrP9MDH5MBPbIqV92AaeXatLxBI9gBaebbnrfifHhDYfgasaacH8akY=wiFfYdH8Gipec8Eeeu0xXdbba9frFj0=OqFfea0dXdd9vqai=hGuQ8kuc9pgc9s8qqaq=dirpe0xb9q8qiLsFr0=vr0=vr0dc8meaabaqaciaacaGaaeqabaqabeGadaaakeaaieqacuWFqbaugaqcaaaa@2DEB@ in such a way that measurements with large uncertainties are given less weight. Once each row has been projected in this way, the estimate of **C **is obtained in the usual way (see Eq. (2)), except using X^
 MathType@MTEF@5@5@+=feaafiart1ev1aaatCvAUfKttLearuWrP9MDH5MBPbIqV92AaeXatLxBI9gBaebbnrfifHhDYfgasaacH8akY=wiFfYdH8Gipec8Eeeu0xXdbba9frFj0=OqFfea0dXdd9vqai=hGuQ8kuc9pgc9s8qqaq=dirpe0xb9q8qiLsFr0=vr0=vr0dc8meaabaqaciaacaGaaeqabaqabeGadaaakeaaieqacuWFybawgaqcaaaa@2DFB@ instead of **X**.

The second half of the ATLS algorithm proceeds in a similar manner except that the maximum likelihood projection into the space of C^
 MathType@MTEF@5@5@+=feaafiart1ev1aaatCvAUfKttLearuWrP9MDH5MBPbIqV92AaeXatLxBI9gBaebbnrfifHhDYfgasaacH8akY=wiFfYdH8Gipec8Eeeu0xXdbba9frFj0=OqFfea0dXdd9vqai=hGuQ8kuc9pgc9s8qqaq=dirpe0xb9q8qiLsFr0=vr0=vr0dc8meaabaqaciaacaGaaeqabaqabeGadaaakeaaieqacuWFdbWqgaqcaaaa@2DD1@ is carried out using the columns of **X **instead of the rows:

x^·j=C^(C^TΨj−1C^)−1C^TΨj−1x·j     (5)
 MathType@MTEF@5@5@+=feaafiart1ev1aaatCvAUfKttLearuWrP9MDH5MBPbIqV92AaeXatLxBI9gBaebbnrfifHhDYfgasaacH8akY=wiFfYdH8Gipec8Eeeu0xXdbba9frFj0=OqFfea0dXdd9vqai=hGuQ8kuc9pgc9s8qqaq=dirpe0xb9q8qiLsFr0=vr0=vr0dc8meaabaqaciaacaGaaeqabaqabeGadaaakeaaieqacuWF4baEgaqcamaaBaaaleaacqWIpM+zcqWGQbGAaeqaaOGaeyypa0Jaf83qamKbaKaacqGGOaakcuWFdbWqgaqcamaaCaaaleqabaGaeeivaqfaaGGabOGae4hQdK1aa0baaSqaaiabdQgaQbqaaiabgkHiTiabigdaXaaakiqb=neadzaajaGaeiykaKYaaWbaaSqabeaacqGHsislcqaIXaqmaaGccuWFdbWqgaqcamaaCaaaleqabaGaeeivaqfaaOGae4hQdK1aa0baaSqaaiabdQgaQbqaaiabgkHiTiabigdaXaaakiab=Hha4naaBaaaleaacqWIpM+zcqWGQbGAaeqaaOGaaCzcaiaaxMaadaqadiqaaiabiwda1aGaayjkaiaawMcaaaaa@517E@

In this case **x**_•*j *_is the *j*th column of **X **and **Ψ**_*j *_is the corresponding error covariance matrix, which is a diagonal matrix consisting of elements from the *j*th column of **S**.

The least squares problem is once again solved using an analog of Eq. (3) employing X^
 MathType@MTEF@5@5@+=feaafiart1ev1aaatCvAUfKttLearuWrP9MDH5MBPbIqV92AaeXatLxBI9gBaebbnrfifHhDYfgasaacH8akY=wiFfYdH8Gipec8Eeeu0xXdbba9frFj0=OqFfea0dXdd9vqai=hGuQ8kuc9pgc9s8qqaq=dirpe0xb9q8qiLsFr0=vr0=vr0dc8meaabaqaciaacaGaaeqabaqabeGadaaakeaaieqacuWFybawgaqcaaaa@2DFB@.

Using the MCR-WALS algorithm, the error structure inherent in the microarray data can be incorporated in the curve resolution procedure. Furthermore, missing data can be accommodated by assigning a very large variance to the associated measurements.

## Results and discussion

### Yeast cell cycle data

To demonstrate the utility of the algorithms proposed here, microarray data related to the cell cycle of *Saccharomyces cerivisiae *described by Spellman *et al*. [32] were employed. Specifically, the subset of data related to the α-factor block release experiment were used. The data in the α-factor release subset consisted of microarray measurements for 6178 open reading frames at 7 minute intervals from 0 to 119 minutes, for a total of 18 experiments. This data set was further screened to exclude any genes for which there were more than 4 missing measurements. This data set will be referred to as "Alpha-full" here and consisted of 6044 genes. In addition to this, a smaller set of 696 pre-selected genes from this group was used as well. These genes, identified as exhibiting cell cycle-dependent changes in *m*RNA expression levels, were the same as those employed by Lu *et al*. [2]. This data set will be referred to as "Alpha-696" in this work. For both data sets, a corresponding measurement standard deviation matrix was constructed by assuming proportional errors of 20% of the measurement. This value is consistent with observations we have made on other microarrays, but is not critical since the absolute magnitude of the proportional error weighting will not affect the results. In addition, missing measurements (2042 in Alpha-full, 0 in Alpha-696) were set to zero and the corresponding error standard deviation was set to a value much greater than the largest proportional error value in the data (a value of 100 was used in this work).

The cell cycle data set was used because it has been widely studied and exhibits some temporally structured patterns of gene expression. In addition, a number of genes postulated to be associated with cell cycle regulation have been identified. It was hoped confirmation of these patterns could be established through curve resolution methods, although a one-to-one correspondence between the underlying regulating factors and the genes related to these cycles is not necessarily required. It should be emphasized, however, that the objective of this work is to demonstrate the utility of the curve resolution method and not to conduct an extensive analysis of the cell cycle using this tool.

### Curve resolution of Alpha-696 data

**Figure 2:**
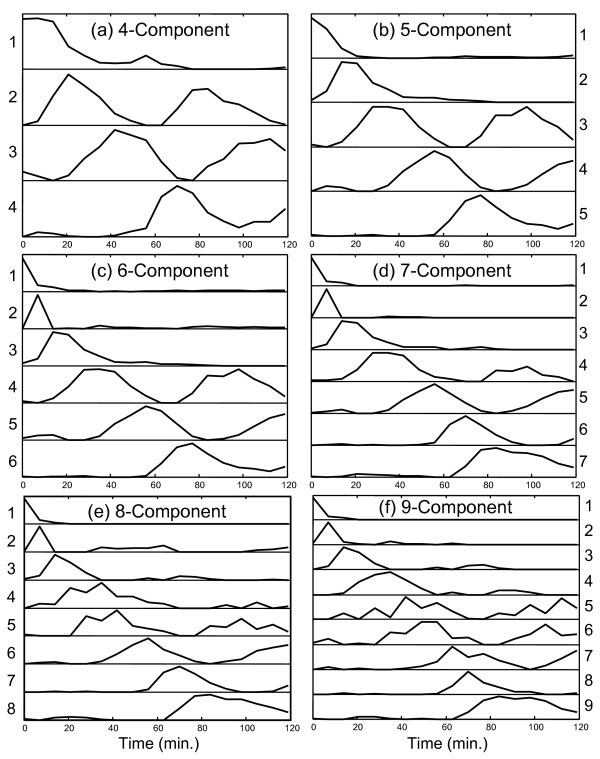
**MCR-WALS results (P matrix) for Alpha-696 data set**. Profile vectors (normalized to unit length) extracted for models with 4 to 9 components are shown. The vectors are arranged in order of appearance of the first major peak in the profile.

Initially the MCR-WALS algorithm was applied to the Alpha-696 data set since this had been prescreened to select for genes with cell-cycle related expression patterns and therefore was thought to be more amenable to successful curve resolution analysis. The data were analyzed by specifying 4, 5, 6, 7, 8 and 9 components (factors) and the extracted time profiles (normalized to unit length) are shown in Figure 2 for each case. Different numbers of components were used because it was not known *a priori *how many underlying factors would be present, although it was suspected that a lower limit would be related to the number of different phases in the cell cycle. Furthermore, more confidence in the results can be achieved if the profiles remain consistent as the number of components is increased. If extraneous components are extracted, these can often be identified by an inconsistent or irregular pattern, or because they correlate with only a few genes that may represent outliers. The profiles extracted by MCR-WALS are not obtained in any particular order, but for the purposes of representation and comparison, they have been arranged in Figure 2 by the order of appearance of the first significant peak value.

The first and most significant feature to notice about Figure 2 is that all of the profiles extracted are consistent with the dynamics of the system being studied. In some cases, a unimodal profile compatible with a unique set of conditions is observed, while in other cases a cyclical pattern suggesting a relationship with the cell cycle is apparent. Most patterns are clear and smooth, with well defined maxima and minima that fall close to zero. These features alone indicate that the results of curve resolution are meaningful and suggest the potential utility of the method. While it is not essential that these underlying regulatory profiles correlate directly with stages in the cell cycle, since the latter are only required to be linear combinations of the former, there is a natural expectation that this will be the case. Because of this, these relationships warrant further investigation.

**Figure 3:**
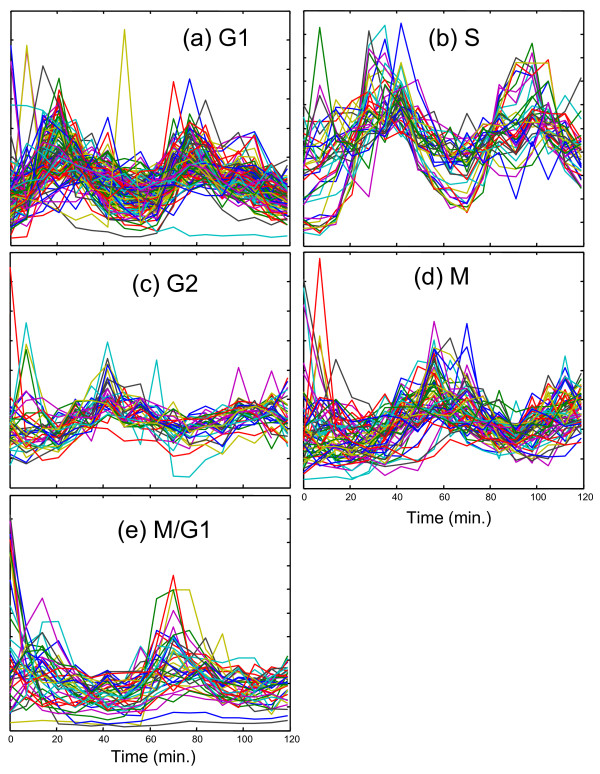
**Profiles of designated cell cycle regulated genes**. Expression profiles (normalized to unit length) for the 292 cell cycle regulated genes identified by Spellman *et al*. [32] are shown grouped by the associated phase.

Considering first the four-component analysis shown in Figure 2a, the relationships with stages of the cell cycle were investigated in two ways. First, the 292 genes classified by Spellman *et al*. (ref [32]; Fig. 7) into five categories related to the yeast cell cycle were extracted. The expression profiles of these genes were then normalized and plotted as shown in Figure 3. This allowed a visual comparison between the profiles extracted and those observed for genes reported to be representative of that stage of the cell cycle. Note that, in this plot, missing measurements were interpolated between the surrounding measurements. A second, more quantitative comparison was made through a correlation study, the results of which are presented in Table 1. This was conducted by first finding the genes in the entire set (Alpha-full, 6044 genes) that correlated most strongly with each of the profiles extracted. Correlation coefficients were calculated around the origin (rather than around the mean) and a cutoff of >0.8 was arbitrarily chosen to indicate substantial similarity with the profiles. The number of genes meeting this criterion for each profile is listed as the parameter "N_tot_" in Table 1. Within this group, the number of genes which were on Spellman's list of 292 genes is was also determined and is given as "(N_match_)" in the table. For each profile, correlated genes that were found on this list are given in the table in descending order of correlation, along with the cell cycle stages to which they were assigned. Also shown in parentheses is the rank of that gene (among all genes) in the correlation list and the corresponding correlation coefficient. In order to conserve space, a maximum number of 20 genes from each list was allowed in the table.

The first of the four curves in Figure 2a (curve 1, as labeled on the left-hand side) is characterized by a maximum at time zero that falls relatively quickly to baseline levels. This curve was initially thought to be associated with genes that are down-regulated after release from α-factor arrest as they do not appear to be part of the regular cell cycle, although there is another small increase around one hour. In Table 1, only 5 of the genes classified in Spellman's list correlate highly with this profile (r>0.8) and only 35 genes in the set of 6044 in Alpha-full show this level of correlation. Interestingly, the most highly correlated profile of the 6044 genes is MFA1, which has been classified as being associated with G1. The other four classified genes are associated with M/G1. The fact that only 35 genes exhibit an expression profile with a correlation of >0.8 suggests that this profile is rarely observed in its pure form, but is likely to be a component of many genes. The significance of this is discussed in more detail in the context of curve 4 below.

The second curve in Figure 2a has a much clearer interpretation. Comparison with Figure 3a readily suggests an association of this profile with G1. This is further supported by the data in Table 1. All of the top five correlated genes have been classified by Spellman as G1, as well as 20 of the top 30. A total of 82 of a possible 119 genes classified as G1 by Spellman have a correlation coefficient of >0.8 with curve 2, while 3 were classified as M/G1 and 7 were classified as S, for a total of 92 genes from Spellman's list. In order to make this classification more quantitative, each profile was given a score for each of the five cell cycle classes. This score was calculated by:

Score(p,c)=∑i=1Ncprip/Nctot     (6)
 MathType@MTEF@5@5@+=feaafiart1ev1aaatCvAUfKttLearuWrP9MDH5MBPbIqV92AaeXatLxBI9gBaebbnrfifHhDYfgasaacH8akY=wiFfYdH8Gipec8Eeeu0xXdbba9frFj0=OqFfea0dXdd9vqai=hGuQ8kuc9pgc9s8qqaq=dirpe0xb9q8qiLsFr0=vr0=vr0dc8meaabaqaciaacaGaaeqabaqabeGadaaakeaacqWGtbWucqWGJbWycqWGVbWBcqWGYbGCcqWGLbqzcqGGOaakcqWGWbaCcqGGSaalcqWGJbWycqGGPaqkcqGH9aqpdaWcgaqaamaaqahabaGaemOCai3aaSbaaSqaaiabdMgaPjabdchaWbqabaaabaGaemyAaKMaeyypa0JaeGymaedabaGaemOta40aaSbaaWqaaiabdogaJjabdchaWbqabaaaniabggHiLdaakeaacqWGobGtdaWgaaWcbaGaem4yamMaemiDaqNaem4Ba8MaemiDaqhabeaaaaGccaWLjaGaaCzcamaabmGabaGaeGOnaydacaGLOaGaayzkaaaaaa@526C@

This equation describes the score for profile *p *on class *c*, where *c *represents G1, S, G2, M or M/G1. The term *r*_*ip *_represents the correlation coefficient between profile *p *and the expression profile of gene *i*, where the summation is over all *N*_*cp *_genes in class *c *(as given by Spellman) that have a correlation coefficient greater than 0.8 with profile *p*. The quantity *N*_*ctot *_is the total number of genes in that as class classified by Spellman (G1 = 119, S = 37, G2 = 34, M = 61, M/G1 = 41). As an example, if all of the 119 G1 genes in Spellman's list had a correlation coefficient of unity with curve 2, the score would be 1, which is the highest value attainable. In Table 1, the score for curve 2 on G1 is 0.539, while the next highest is 0.154 for S, supporting the classification of G1.

Curve 3 in the four-component case also exhibits a cyclical pattern that is shifted later in time from Curve 2. This would be consistent with S, G2 or M and all of these classes give high scores with Curve 3, with 31/37 of the designated S genes, 27/34 of the designated G2 genes, and 38/61 of the designated M genes giving correlation coefficients above 0.8. Visually (see Figure 3b) and by score, the best match appears to be S, but it is likely that these three groups have merged together in this profile since an inadequate number of components were specified to capture all of the elements of the cell cycle. This is not a very specific group, since more than half of the 6044 genes give a correlation of 0.8 or better.

The fourth curve in Figure 2a gives a poor score with all of the designated classes except for M/G1. Of the 15 highly correlated genes with designated classes, 8 of these are M/G1, 6 are M, and 1 is G1. The score and number of correlated M/G1 genes is not especially high and the reason for this becomes clear on visual inspection of Figure 3. In addition to the peak around 70 minutes, the M/G1 designated genes also exhibit a peak just before the first peak of Curve 2, which has been designated G1. Thus, it would appear that the M/G1 phase is a composite of the two underlying functions shown in curves 1 and 4. Furthermore, close examination of the expression patterns for the designated M/G1 genes in Figure 3e reveals that some of these gene expression profiles are dominated by the first peak, some by the second peak, and some show a distinctive rapid decay from time zero. This suggests the presence of two or more processes underlying these genes. This does not mean that all of these genes cannot be considered to belong to the M/G1 classification, only that there may be more than one driving force behind the expression of these genes.

This initial four-component analysis was promising, but suggested (as anticipated) that there were too few components to adequately model the cell cycle. It was expected that extension to five components would allow better resolution of the S, G2 and M phases. While this did happen, other unexpected observations were made. The results of this analysis are shown in Figure 2b and Table 2 (the correlation coefficients have been removed to save space). In this case, curves 3 and 4 exhibit clear and excellent matches to designated S and M genes, respectively, although curve 3 also exhibits some strong G1 and M character, as might be expected. Also, as for the four-component model, the M/G1 phase seems to be a combination of the first and last curves and therefore gives only a moderate score in each case. What is particularly interesting is that the strongly correlated G1 pattern that was so apparent in the four-component model has disappeared in the five-component case. Instead, in this case, curve 2 represents the first peak of the G1 profile, but because the second cycle is absent, strong correlations with the designated genes are not observed. It is our interpretation that the second cycle of the G1 phase is now represented in curve 5 and what is seen in curves 2 and 5 are two separate regulating profiles for a mix of genes designated as G1 and M/G1. Note that in the five-component case relative to the four-component case, curve 1 falls off more sharply, curve 2 is shifted to an earlier time, and curve 5 (corresponding to curve 4 in Figure 2a) is shifted to a later time, all of which would be consistent with a blending of G1 and M/G1. Another notable feature of the five-component analysis is that there is no clearly defined curve for G2. However, the profiles for the designated G2 genes shown Figure 3c do not show distinctive features and could easily be obtained through linear combinations of the five curves presented.

The six-component model, presented in Figure 2c shows essentially the same features as the five-component model, with the addition of one new profile characterized by a single spike in expression levels at the second time point at t = 7 minutes. Because of the transient nature of this peak, it is not clear whether this represents a real profile, or whether it is simply an artifact of outliers in the data. Only one of the genes in Spellman's classification (PHD1 - M) shows a strong correlation with this profile (rank = 8, r = 0.84) and there are only 10 genes with a correlation above 0.8. This does not, however, exclude it from being a component of other expression profiles. Because the remaining profiles were so similar to the five-component model, a full table of correlation data is not included.

The profiles of the seven-component model shown in Figure 2d includes many of the same patterns as were seen in the five- and six-component models, but also brings a refinement that validates some of the hypotheses extended for the simpler models. To conserve space, the full table of results for the seven-component model is included as supplementary data (see additional file 1: TableA) and only summarized here. Curves 1, 2 and 3 are essentially unchanged from the six-component model. Curve 4, which is associated with the S phase, has a notable change in that the second cycle is considerably reduced in its magnitude. The score on the S phase genes is reduced to 0.281, compared to 0.743 and 0.721 for the five- and six-component models, respectively. More importantly, however, this profile is now much more specific for the S phase genes. In the five- and six-component models, the scores of this curve on G1 were 0.326 and 0.305, while the scores on G2 were both 0.443. For the corresponding curve in the seven-component model, the G1 score is only 0.041 and the G2 score is 0. Furthermore, the number of correlated genes has dropped from 1398 in the five-component model and 1294 in the six-component model to only 45 in the seven-component model. This clearly indicates that the model expansion has permitted this curve to become much more specific in representing the S phase.

Curve 5 for the seven-component model, which is representative of the M phase, has remained essentially the same as in the five- and six-component models, but curves 6 and 7 represent a further refinement of the last profile in the previous models. In earlier models, it was postulated that the G1 and M/G1 phases were driven by two distinct underlying functions, each correlated with one of the two peaks in the cycles. For the five-component model, it appeared that the last profile was a blending of the second cycle of these two phases. Now, in the seven-component model, it appears that this mixing has been resolved in curves 6 and 7. Curve 6, which has been shifted to shorter times, is representative of the second cycle of M/G1 and correlates with four designated genes in that group (see additional file 1: TableA). Curve 7 is shifted to longer times and correlates well with the second cycle of the G1 phase, again matching four genes in that group. As before, neither of these curves by itself gives a strong correlation with a large number of designated genes in the corresponding phases, but this is because they only represent half of the cycle. Even so, the genes that are correlated represent a statistically meaningful group of the overall population. When the time profile is divided into two and each half is considered separately, the G1 score for curve 3 increases from 0.007 to 0.405 (first half) and the G1 score for curve 7 increases from 0.028 to 0.870 (second half). Likewise, the M/G1 score for curve 1 increases from 0.130 to 0.161 (first half) and that for curve 6 increases from 0.087 to 0.303 (second half). This is further evidence of two independent driving functions for G1 and M/G1, one which is active on release from α-factor arrest and another which becomes activated in the second cycle.

Extensions of the model to 8 and 9 components, as shown in Figures 2e and 2f, retain essentially the same features as the earlier models, but some more subtle changes are evident. In particular, the profile associated with the S phase (curve 4 in Figures 2c–f) seems to follow the same pattern as the G1 and M/G1 genes, with the two parts of the cycles separating from one another. For the eight-component model, the second half of the S phase is likely modeled by the newly appearing curve 5, which has a high score on the genes classified as S (0.529) as well as a high score on the G2 genes (0.270). For the nine-component model, curve five develops more G2 character and for the first time a profile is classified in this group with a score of 0.470, although it also exhibits similarity to S and M (scores of 0.199 and 0.304, respectively). In this case, some of the second half of the S cycle has likely been blended into the second half of the G1 cycle represented as curve 9. The nine-component model is also characterized by a new profile in curve 6 (although it is arguable which is "new") that appears to be a combination of G2 and M.

At this point, the capabilities of curve resolution are approaching their limits and become increasingly speculative. As more components are added to the model, the algorithm diverts its efforts to modeling more subtle changes in the expression patterns and eventually artifacts that may be more related to noise than biological change creep into the profiles. This is evidenced by some of the later models which show more sporadic variations than the earlier ones. Also, as the changes being modeled in the cell approach finer and finer resolution in the time domain, our confidence in the results becomes eroded. For example, we can question whether curve 2 in Figures 2c–f is real or is an artifact of that particular time point. The limits of the curve resolution approach can be extended in several ways that include the acquisition of better quality data, more frequent sampling of the system, and the inclusion of reliable uncertainty information in the measurements.

**Figure 4:**
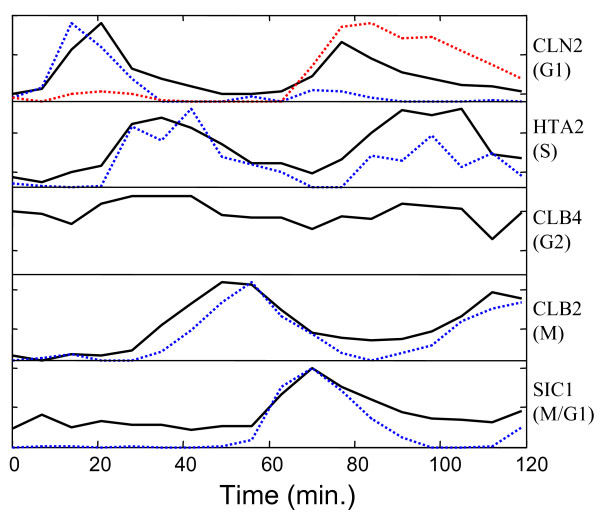
**Comparison of MCR-WALS extracted profiles with designated cell cycle regulated genes**. Selected profile vectors (dashed lines) extracted from the Alpha-696 data set with the 8-component model are compared with the time profiles for representative genes (solid lines) selected by Lu *et al*. [2] for each phase of the cell cycle. Both sets of profiles are normalized to unit length. Two extracted profiles were necessary to account for each cycle of the G1 phase and no clear match was indicated for G2.

It is important to remember that this modeling was performed with no prior assumptions about the components present, only assumptions of bilinearity and non-negativity were used. To further illustrate the effectiveness of curve resolution for extracting cell cycle related information, Figure 4 shows the normalized expression profiles of five genes used by Lu *et al *[2] to represent the phases of the cell cycle. These are shown as solid lines. Superimposed on these (dashed lines) are selected curves from the eight-component model with close matches. For G1, two curves are shown because it is postulated here that this cycle is driven by two different underlying processes. (The same is proposed for M/G1, but only one profile is evident in the selected gene.) No model profile is compared to the selected curve for G2, since there was no definitive match. Overall, the synchronicity of the extracted profiles with the independently selected genes is very good and supports this method of analysis.

**Figure 5:**
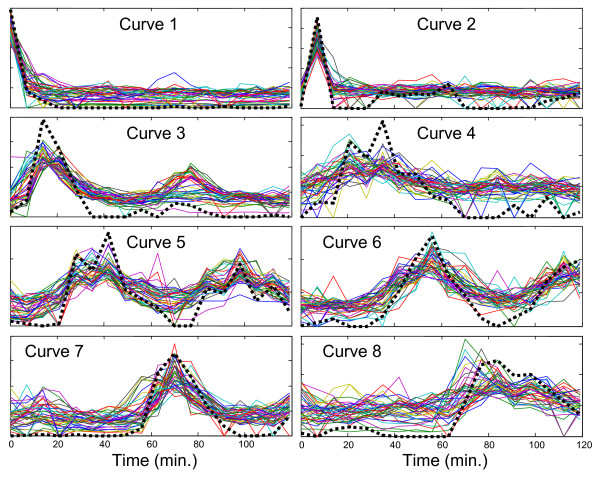
**Comparison of MCR-WALS extracted profiles with highly correlated gene expression profiles**. Profile vectors extracted from the Alpha-696 data set with the 8-component model (thick dashed lines) are compared with the 40 most highly correlated gene expression profiles from the Alpha-full data set. All profiles are normalized to unit length.

As further evidence of the legitimacy of the profiles extracted by curve resolution, Figure 5 shows each of the curves extracted from the eight-component model (dashed lines) plotted with the 40 most highly correlated expression profiles (normalized to unit length) from Alpha-full. This plot confirms the presence of the predominantly unimodal profiles (curves 1,2,3, 4, 7 and 8), supporting the case for separate underlying regulatory factors for G1 and M/G1.

### Curve resolution for Alpha-full data

**Figure 6:**
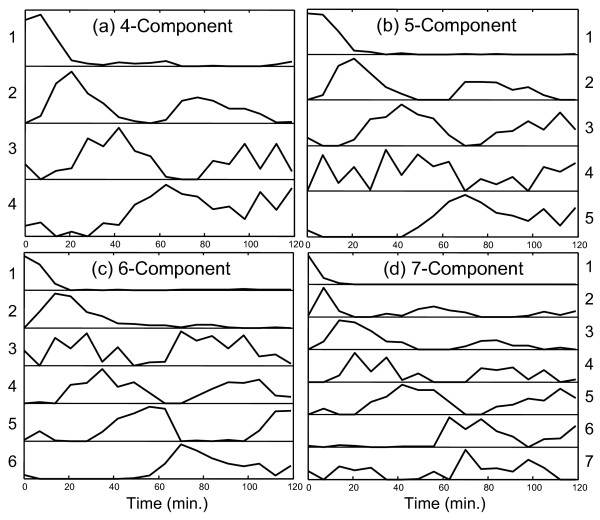
**MCR-ATLS results (P matrix) for Alpha-full data set**. Profile vectors (normalized to unit length) extracted for models with 4 to 7 components are shown. The vectors are arranged in order of appearance of the first major peak in the profile.

In the analysis of the subset of genes represented by the Alpha-696 data set, it could be argued that the original microarray data had already been screened for genes that are known to be associated with cell cycle regulation. In other studies where such a subset selection may not be possible, the utility of the curve resolution method remains to be demonstrated. In other words, can curve resolution be used to extract original information from microarray data sets with no prior knowledge of gene association? To answer this question, the curve resolution algorithm was applied to the entire Alpha-full data set (6044 genes) without any prior information other than the proposed number of components and the non-negativity constraints normally applied. The results of this analysis are presented in Figure 6. The correlation and classification analysis for the four-component model is given in Table 3, and that for the five-component model is included as supplementary data to conserve space (see additional file 2: TableB).

On initial examination of Figure 6, it is immediately clear that the profiles extracted have some association with the cell cycle. Furthermore, comparison with Figure 2 reveals strong similarities in the profiles, although the profiles extracted from the Alpha-696 data set are generally more cleanly defined. This was anticipated, since the cell cycle genes in the expanded data set are diluted by other genes that may be unrelated or noisy. Another difference between the two sets of results is that, while the profiles exhibited are similar, they do not always appear in the same sequence. For example, in the Alpha-696 set, the two cycles of G1 separate into different components by the five-component model, while for the Alpha-full set this does not happen until six components are employed. Likewise, in the full data set, the sharp transient at 7 minutes does not appear until the seven-component model, where it was evident in the six-component model for the reduced data set. This is expected, since the distribution of gene expression profiles will be different in the full data set compared to the reduced set.

A complete analysis of the Alpha-Full profiles will not be carried out here, since the treatment is similar to the reduced data set. The tables show correlation data with the genes classified by Spellman for the four- and five-component models, respectively. For the four-component model (Table 3), the classifications for G1, S, M, and M/G1 (early expression) are clear. For the five-component data (see additional file 2: TableB) the classifications are less definitive, but the profiles still show a strong association with cell cycle regulated genes. In general, the correlation scores become more ambiguous as the number of components increases. This is due to several factors, including the blending of similar profiles, the resolution of profiles into early and late components, and the noise in the profiles resulting from noisy data. Nevertheless, the trends are clear and support the contention that this method can be used to extract underlying information in an unbiased way with no prior knowledge about the data.

### Uniqueness of MCR-WALS solutions

An important consideration in the application of MCR is the uniqueness of the solutions it produces. In the work presented here, one set of solutions was presented for each model/data set combination. This is a common practice in the presentation of MCR results, but it is not very realistic. While it is hoped that the reported solution is representative, a range of equivalent or nearly equivalent solutions is usually possible. Reasons for this include: (1) the possible existence of mathematically degenerate solutions to Eq. (1) (rotational ambiguity), (2) computational and numerical limitations of the method used, and (3) noise in the data.

In their original work on self-modeling curve resolution for two-component systems, Lawton and Sylvestre [19] recognized that a *set *of solutions was possible, even in the presence of constraints. Therefore, the profiles (absorbance spectra in their case) were presented in the form of allowed boundaries ("feasible solutions") where the constraints could be satisfied. Although a number of attempts have been made to extend the analytical solution provided by Lawton and Sylvestre to more than two components [33-35], this has proven to be very difficult, especially in the presence of noisy data. Moreover, the notion of profile "boundaries" is not very meaningful in higher dimensions, since all of the profiles in a degenerate set are linked together and this is not reflected in the presentation of profile boundaries; *i.e*. it is not possible to mix solutions for the components arbitrarily [36]. Some attempts have been made to attach boundaries to modern MCR methods such as ALS with mixed success [36-38]. Fortunately, many experimental situations lead to solutions that are unique or tightly bounded, so a single solution is often acceptable. This is because the nature of the data may lead to measurement points (*e.g*. times or genes) that are unique or highly selective for one component. Unfortunately, this is difficult to assess *a priori*.

Another source of multiple solutions is computational limitations. It is possible (and likely) that different starting points will yield different solutions, not only because of rotational ambiguities, but also because of local minima or premature termination. The ALS algorithm is quite stable in its convergence properties (although it can be slower than other minimization strategies) and this is one of the reasons for its popularity, but it is not immune from numerical problems. In this work, the use of the SIMPLISMA algorithm [22] removed the random element of initialization, although it should be noted that this method does not work well in the presence of large amounts of noise.

Finally, measurement noise plays an important role in the solutions obtained. Clearly, the data represents a single realization of the experimental results and MCR solutions for replicate experiments are not expected to be identical. Without the availability of replicate data, this contribution to the variability is difficult to assess directly, but it can be inferred through re-sampling methods.

**Figure 7:**
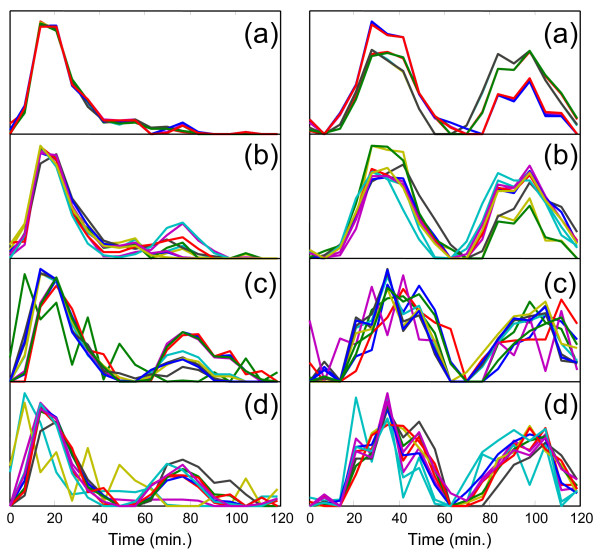
**Reproducibility of MCR-WALS results for two representative profiles from the six-component model**. Profile vectors for two selected components of the six-component model extracted under different conditions. In each case, ten replicate runs were made. (a) Alpha 696, random initialization, (b) Alpha-696, random subsampling, (c) Alpha-full, random initialization, (d) Alpha-full, random subsampling.

To gain insight into the reproducibility of the solutions presented in Figures 2 and 6, two approaches, "random initialization" and "random sub-sampling" were adopted and new solutions were generated from ten replicate runs of the MCR-WALS algorithm in each case. In the random initialization approach, different initial estimates of **P **were obtained each time the program was run by randomly selecting individual gene profiles that were used as starting values. In the random sub-sampling approach, ten subsets of data, each half the size of the original data set, were obtained by randomly selecting gene expression profiles from the original data. These were then analyzed by MCR-WALS with initial estimates obtained via SIMPLISMA. Figure 7 shows some of the results of these studies. Although models with fewer components generally produce more reproducible results, the profiles in Figure 7 are for the more demanding six-component model. For simplicity, only two components were chosen for display and these were picked on the basis of their consistency among different models and between the Alpha-696 and Alpha-full data sets (components 4/4 and 3/2 in Figures 2/6).

The results in Figure 7 show good reproducibility among the profiles extracted in terms of the major features that they exhibit. As expected, the range of solutions is generally narrower for the Alpha-696 data, since these genes were preselected on the basis of cell-cycle association, but good results were still obtained for the full set of genes. For the bimodal curves in the right-hand panels, there is some shifting of the relative contributions of the two peaks and some small changes in their positions, but the association of these profiles with the cell cycle is unmistakable and is clearly not a statistical aberration. In the left-hand panels, a feature that was not apparent in either of the original analyses but was evident in the reproducibility studies is the second peak that occurs around 80 min. The presence of this peak is consistent with the second cycle of G1-regulated genes that were associated with the unimodal peak in the original analysis, so its appearance is not surprising and supports the original classification.

Clearly this type of reproducibility analysis is useful in assessing the reliability of the profiles extracted by MCR-WALS. Although there might be an inclination to report the average of these solutions as an overall solution, this is not recommended since the average profiles do not generally define an acceptable solution set. It should be noted that some of the profiles in the higher component models showed significant variability, but this was expected and is intimated at by the shape of the profiles themselves. In addition, obtaining consistent matching of the profiles from replicate runs can be a challenge, since the correlations are not always obvious.

## Conclusion

The primary objective of this work has been to demonstrate that the MCR-WALS algorithm is an effective tool for extracting useful information from serial microarray experiments. Features of the method include (1) it is relatively simple and efficient, (2) it makes no assumptions about the underlying model other than linearity and non-negativity in the contribution and profile matrices, (3) it is applicable to untransformed expression data, and (4) it can accommodate arbitrary error structures and missing data (within reasonable limits). Through the application of MCR-WALS to yeast cell cycle data, we have demonstrated the utility of the profile vectors in the interpretation of gene expression regulation. With no prior information, the algorithm was able to extract profiles that were clearly associated with cell cycle regulated genes, even when the full data set was used. Moreover, the results indicated the possibility of more than one underlying regulatory factor in some cases, suggesting that this approach could be a valuable tool in the inferential study of cellular regulation.

More work needs to be done to establish the utility of this approach and expand its capabilities. This includes further validation of the MCR-WALS algorithm through its application to other experiments and the development of better methods to interpret the profile and contribution matrices in a biological context. At present, the complexities of biological models for gene regulation make it difficult to establish a direct physical relationship to the linear model used in this work, although this would clearly be useful. The "components" or "factors" used here are assumed to have some association with regulatory factors in the cell, but it is likely that limitations in the experimental measurements restrict the number of regulatory inputs that can be reliably modeled. Nevertheless, the profiles extracted and their relationships to the expression of individual genes should serve as a starting point for more extensive investigation. Further algorithmic improvements, such as the inclusion of additional biologically relevant constraints on the solutions and the development of methods to better estimate the number of factors, should also improve the utility of the methodology.

## Methods

The MCR-WALS algorithm used in this work is presented below.

1. Choose the number of components, *p*, and the initial estimate for **P**, designated as P^
 MathType@MTEF@5@5@+=feaafiart1ev1aaatCvAUfKttLearuWrP9MDH5MBPbIqV92AaeXatLxBI9gBaebbnrfifHhDYfgasaacH8akY=wiFfYdH8Gipec8Eeeu0xXdbba9frFj0=OqFfea0dXdd9vqai=hGuQ8kuc9pgc9s8qqaq=dirpe0xb9q8qiLsFr0=vr0=vr0dc8meaabaqaciaacaGaaeqabaqabeGadaaakeaaieqacuWFqbaugaqcaaaa@2DEB@. Normalize the row vectors of P^
 MathType@MTEF@5@5@+=feaafiart1ev1aaatCvAUfKttLearuWrP9MDH5MBPbIqV92AaeXatLxBI9gBaebbnrfifHhDYfgasaacH8akY=wiFfYdH8Gipec8Eeeu0xXdbba9frFj0=OqFfea0dXdd9vqai=hGuQ8kuc9pgc9s8qqaq=dirpe0xb9q8qiLsFr0=vr0=vr0dc8meaabaqaciaacaGaaeqabaqabeGadaaakeaaieqacuWFqbaugaqcaaaa@2DEB@ to unit length. Estimate a matrix of error variances for **X**, designated as **S**.

2. Perform a maximum likelihood projection of each row of **X **into the space of P^
 MathType@MTEF@5@5@+=feaafiart1ev1aaatCvAUfKttLearuWrP9MDH5MBPbIqV92AaeXatLxBI9gBaebbnrfifHhDYfgasaacH8akY=wiFfYdH8Gipec8Eeeu0xXdbba9frFj0=OqFfea0dXdd9vqai=hGuQ8kuc9pgc9s8qqaq=dirpe0xb9q8qiLsFr0=vr0=vr0dc8meaabaqaciaacaGaaeqabaqabeGadaaakeaaieqacuWFqbaugaqcaaaa@2DEB@ to give an estimated X^
 MathType@MTEF@5@5@+=feaafiart1ev1aaatCvAUfKttLearuWrP9MDH5MBPbIqV92AaeXatLxBI9gBaebbnrfifHhDYfgasaacH8akY=wiFfYdH8Gipec8Eeeu0xXdbba9frFj0=OqFfea0dXdd9vqai=hGuQ8kuc9pgc9s8qqaq=dirpe0xb9q8qiLsFr0=vr0=vr0dc8meaabaqaciaacaGaaeqabaqabeGadaaakeaaieqacuWFybawgaqcaaaa@2DFB@:

x^i·=xi·Σi−1P^T(P^Σi−1P^T)−1P^     (7)
 MathType@MTEF@5@5@+=feaafiart1ev1aaatCvAUfKttLearuWrP9MDH5MBPbIqV92AaeXatLxBI9gBaebbnrfifHhDYfgasaacH8akY=wiFfYdH8Gipec8Eeeu0xXdbba9frFj0=OqFfea0dXdd9vqai=hGuQ8kuc9pgc9s8qqaq=dirpe0xb9q8qiLsFr0=vr0=vr0dc8meaabaqaciaacaGaaeqabaqabeGadaaakeaaieqacuWF4baEgaqcamaaBaaaleaacqWGPbqAcqWIpM+zaeqaaOGaeyypa0Jae8hEaG3aaSbaaSqaaiabdMgaPjabl+y6NbqabaacceGccqGFJoWudaqhaaWcbaGaemyAaKgabaGaeyOeI0IaeGymaedaaOGaf8huaaLbaKaadaahaaWcbeqaaiabbsfaubaakiabcIcaOiqb=bfaqzaajaGae43Odm1aa0baaSqaaiabdMgaPbqaaiabgkHiTiabigdaXaaakiqb=bfaqzaajaWaaWbaaSqabeaacqqGubavaaGccqGGPaqkdaahaaWcbeqaaiabgkHiTiabigdaXaaakiqb=bfaqzaajaGaaCzcaiaaxMaadaqadiqaaiabiEda3aGaayjkaiaawMcaaaaa@51CC@

where x^
 MathType@MTEF@5@5@+=feaafiart1ev1aaatCvAUfKttLearuWrP9MDH5MBPbIqV92AaeXatLxBI9gBaebbnrfifHhDYfgasaacH8akY=wiFfYdH8Gipec8Eeeu0xXdbba9frFj0=OqFfea0dXdd9vqai=hGuQ8kuc9pgc9s8qqaq=dirpe0xb9q8qiLsFr0=vr0=vr0dc8meaabaqaciaacaGaaeqabaqabeGadaaakeaaieqacuWF4baEgaqcaaaa@2E3B@_*i*• _represents the *i*th row vector of **X **and **Σ**_*i *_is a diagonal matrix formed from the *i*th row of **S**.

3. Solve X^
 MathType@MTEF@5@5@+=feaafiart1ev1aaatCvAUfKttLearuWrP9MDH5MBPbIqV92AaeXatLxBI9gBaebbnrfifHhDYfgasaacH8akY=wiFfYdH8Gipec8Eeeu0xXdbba9frFj0=OqFfea0dXdd9vqai=hGuQ8kuc9pgc9s8qqaq=dirpe0xb9q8qiLsFr0=vr0=vr0dc8meaabaqaciaacaGaaeqabaqabeGadaaakeaaieqacuWFybawgaqcaaaa@2DFB@ = C^
 MathType@MTEF@5@5@+=feaafiart1ev1aaatCvAUfKttLearuWrP9MDH5MBPbIqV92AaeXatLxBI9gBaebbnrfifHhDYfgasaacH8akY=wiFfYdH8Gipec8Eeeu0xXdbba9frFj0=OqFfea0dXdd9vqai=hGuQ8kuc9pgc9s8qqaq=dirpe0xb9q8qiLsFr0=vr0=vr0dc8meaabaqaciaacaGaaeqabaqabeGadaaakeaaieqacuWFdbWqgaqcaaaa@2DD1@P^
 MathType@MTEF@5@5@+=feaafiart1ev1aaatCvAUfKttLearuWrP9MDH5MBPbIqV92AaeXatLxBI9gBaebbnrfifHhDYfgasaacH8akY=wiFfYdH8Gipec8Eeeu0xXdbba9frFj0=OqFfea0dXdd9vqai=hGuQ8kuc9pgc9s8qqaq=dirpe0xb9q8qiLsFr0=vr0=vr0dc8meaabaqaciaacaGaaeqabaqabeGadaaakeaaieqacuWFqbaugaqcaaaa@2DEB@ + **E **for C^
 MathType@MTEF@5@5@+=feaafiart1ev1aaatCvAUfKttLearuWrP9MDH5MBPbIqV92AaeXatLxBI9gBaebbnrfifHhDYfgasaacH8akY=wiFfYdH8Gipec8Eeeu0xXdbba9frFj0=OqFfea0dXdd9vqai=hGuQ8kuc9pgc9s8qqaq=dirpe0xb9q8qiLsFr0=vr0=vr0dc8meaabaqaciaacaGaaeqabaqabeGadaaakeaaieqacuWFdbWqgaqcaaaa@2DD1@ given X^
 MathType@MTEF@5@5@+=feaafiart1ev1aaatCvAUfKttLearuWrP9MDH5MBPbIqV92AaeXatLxBI9gBaebbnrfifHhDYfgasaacH8akY=wiFfYdH8Gipec8Eeeu0xXdbba9frFj0=OqFfea0dXdd9vqai=hGuQ8kuc9pgc9s8qqaq=dirpe0xb9q8qiLsFr0=vr0=vr0dc8meaabaqaciaacaGaaeqabaqabeGadaaakeaaieqacuWFybawgaqcaaaa@2DFB@ and P^
 MathType@MTEF@5@5@+=feaafiart1ev1aaatCvAUfKttLearuWrP9MDH5MBPbIqV92AaeXatLxBI9gBaebbnrfifHhDYfgasaacH8akY=wiFfYdH8Gipec8Eeeu0xXdbba9frFj0=OqFfea0dXdd9vqai=hGuQ8kuc9pgc9s8qqaq=dirpe0xb9q8qiLsFr0=vr0=vr0dc8meaabaqaciaacaGaaeqabaqabeGadaaakeaaieqacuWFqbaugaqcaaaa@2DEB@. Use NNLS, or truncate the negative elements to zero.

4. Perform a maximum likelihood projection of each column of **X **into the space of c^
 MathType@MTEF@5@5@+=feaafiart1ev1aaatCvAUfKttLearuWrP9MDH5MBPbIqV92AaeXatLxBI9gBaebbnrfifHhDYfgasaacH8akY=wiFfYdH8Gipec8Eeeu0xXdbba9frFj0=OqFfea0dXdd9vqai=hGuQ8kuc9pgc9s8qqaq=dirpe0xb9q8qiLsFr0=vr0=vr0dc8meaabaqaciaacaGaaeqabaqabeGadaaakeaaieqacuWFJbWygaqcaaaa@2E11@ to give an estimated X^
 MathType@MTEF@5@5@+=feaafiart1ev1aaatCvAUfKttLearuWrP9MDH5MBPbIqV92AaeXatLxBI9gBaebbnrfifHhDYfgasaacH8akY=wiFfYdH8Gipec8Eeeu0xXdbba9frFj0=OqFfea0dXdd9vqai=hGuQ8kuc9pgc9s8qqaq=dirpe0xb9q8qiLsFr0=vr0=vr0dc8meaabaqaciaacaGaaeqabaqabeGadaaakeaaieqacuWFybawgaqcaaaa@2DFB@:

x^·j=C^(C^TΨj−1C^)−1C^TΨj−1x·j     (8)
 MathType@MTEF@5@5@+=feaafiart1ev1aaatCvAUfKttLearuWrP9MDH5MBPbIqV92AaeXatLxBI9gBaebbnrfifHhDYfgasaacH8akY=wiFfYdH8Gipec8Eeeu0xXdbba9frFj0=OqFfea0dXdd9vqai=hGuQ8kuc9pgc9s8qqaq=dirpe0xb9q8qiLsFr0=vr0=vr0dc8meaabaqaciaacaGaaeqabaqabeGadaaakeaaieqacuWF4baEgaqcamaaBaaaleaacqWIpM+zcqWGQbGAaeqaaOGaeyypa0Jaf83qamKbaKaacqGGOaakcuWFdbWqgaqcamaaCaaaleqabaGaeeivaqfaaGGabOGae4hQdK1aa0baaSqaaiabdQgaQbqaaiabgkHiTiabigdaXaaakiqb=neadzaajaGaeiykaKYaaWbaaSqabeaacqGHsislcqaIXaqmaaGccuWFdbWqgaqcamaaCaaaleqabaGaeeivaqfaaOGae4hQdK1aa0baaSqaaiabdQgaQbqaaiabgkHiTiabigdaXaaakiab=Hha4naaBaaaleaacqWIpM+zcqWGQbGAaeqaaOGaaCzcaiaaxMaadaqadiqaaiabiIda4aGaayjkaiaawMcaaaaa@5184@

5. Solve X^
 MathType@MTEF@5@5@+=feaafiart1ev1aaatCvAUfKttLearuWrP9MDH5MBPbIqV92AaeXatLxBI9gBaebbnrfifHhDYfgasaacH8akY=wiFfYdH8Gipec8Eeeu0xXdbba9frFj0=OqFfea0dXdd9vqai=hGuQ8kuc9pgc9s8qqaq=dirpe0xb9q8qiLsFr0=vr0=vr0dc8meaabaqaciaacaGaaeqabaqabeGadaaakeaaieqacuWFybawgaqcaaaa@2DFB@ = C^
 MathType@MTEF@5@5@+=feaafiart1ev1aaatCvAUfKttLearuWrP9MDH5MBPbIqV92AaeXatLxBI9gBaebbnrfifHhDYfgasaacH8akY=wiFfYdH8Gipec8Eeeu0xXdbba9frFj0=OqFfea0dXdd9vqai=hGuQ8kuc9pgc9s8qqaq=dirpe0xb9q8qiLsFr0=vr0=vr0dc8meaabaqaciaacaGaaeqabaqabeGadaaakeaaieqacuWFdbWqgaqcaaaa@2DD1@P^
 MathType@MTEF@5@5@+=feaafiart1ev1aaatCvAUfKttLearuWrP9MDH5MBPbIqV92AaeXatLxBI9gBaebbnrfifHhDYfgasaacH8akY=wiFfYdH8Gipec8Eeeu0xXdbba9frFj0=OqFfea0dXdd9vqai=hGuQ8kuc9pgc9s8qqaq=dirpe0xb9q8qiLsFr0=vr0=vr0dc8meaabaqaciaacaGaaeqabaqabeGadaaakeaaieqacuWFqbaugaqcaaaa@2DEB@ + **E **for P^
 MathType@MTEF@5@5@+=feaafiart1ev1aaatCvAUfKttLearuWrP9MDH5MBPbIqV92AaeXatLxBI9gBaebbnrfifHhDYfgasaacH8akY=wiFfYdH8Gipec8Eeeu0xXdbba9frFj0=OqFfea0dXdd9vqai=hGuQ8kuc9pgc9s8qqaq=dirpe0xb9q8qiLsFr0=vr0=vr0dc8meaabaqaciaacaGaaeqabaqabeGadaaakeaaieqacuWFqbaugaqcaaaa@2DEB@ given X^
 MathType@MTEF@5@5@+=feaafiart1ev1aaatCvAUfKttLearuWrP9MDH5MBPbIqV92AaeXatLxBI9gBaebbnrfifHhDYfgasaacH8akY=wiFfYdH8Gipec8Eeeu0xXdbba9frFj0=OqFfea0dXdd9vqai=hGuQ8kuc9pgc9s8qqaq=dirpe0xb9q8qiLsFr0=vr0=vr0dc8meaabaqaciaacaGaaeqabaqabeGadaaakeaaieqacuWFybawgaqcaaaa@2DFB@ and C^
 MathType@MTEF@5@5@+=feaafiart1ev1aaatCvAUfKttLearuWrP9MDH5MBPbIqV92AaeXatLxBI9gBaebbnrfifHhDYfgasaacH8akY=wiFfYdH8Gipec8Eeeu0xXdbba9frFj0=OqFfea0dXdd9vqai=hGuQ8kuc9pgc9s8qqaq=dirpe0xb9q8qiLsFr0=vr0=vr0dc8meaabaqaciaacaGaaeqabaqabeGadaaakeaaieqacuWFdbWqgaqcaaaa@2DD1@. Use NNLS, or truncate the negative elements to zero. Normalize the rows of P^
 MathType@MTEF@5@5@+=feaafiart1ev1aaatCvAUfKttLearuWrP9MDH5MBPbIqV92AaeXatLxBI9gBaebbnrfifHhDYfgasaacH8akY=wiFfYdH8Gipec8Eeeu0xXdbba9frFj0=OqFfea0dXdd9vqai=hGuQ8kuc9pgc9s8qqaq=dirpe0xb9q8qiLsFr0=vr0=vr0dc8meaabaqaciaacaGaaeqabaqabeGadaaakeaaieqacuWFqbaugaqcaaaa@2DEB@ to unit length.

6. Repeat from Step 2 until convergence.

This algorithm was implemented in MatLab^® ^(The MathWorks, Natick, MA) under Microsoft Windows^® ^platform. MatLab^® ^programs (".m" text files) and data sets (".mat" files) used in this work are available for download from the corresponding author's web-site. [39]. The data used are also available in a standard spreadsheet format as supplementary material (see additional file 3:SpelData). The program made available uses simple truncation of the least squares solution as opposed to NNLS and provides three options for initialization (random initialization, random subsets, and SIMPLISMA). As noted, the use of different starting profiles can result in small differences in the results obtained, but the same patterns should be observed in all cases. For the results reported here, the SIMPLISMA method was used.

## Authors' contributions

PDW and TKK carried out the data analysis and wrote the draft manuscript. MWW, SR, MJM and CPA were involved in the conception of the study, the interpretation of the results and the revision of the manuscript.

## Supplementary Material

Additional File 1 Analysis of 7-component curve resolution results from Alpha-696 data. Click here for file

Additional File 2 Analysis of 5-component curve resolution results from Alpha-full data. Click here for file

Additional File 3 Data used in the curve resolution analysis. File includes the gene IDs, ratios, and estimated ratio standard deviations for the Alpha-696 and Alpha-full data sets as used in this work. Also included are the gene classifications reported by Spellman and the gene name list employed. Click here for file

## Figures and Tables

**Table 1:** Analysis of 4-component curve resolution results from Alpha-696 data. Cell-cycle related genes (as classified by Spellman [32]) from Alpha-full (6044 genes) that have a strong correlation (r>0.8) with extracted profiles are listed, along with the associated phase. The numbers in parentheses give the ranking in the correlation list and the correlation coefficient. See text for more details.

	Curve 1	Curve 2	Curve 3	Curve 4
	MFA1 **G1 **(1, 0.96)	SVS1 **G1 **(1, 0.98)	ALK1 **M **(2, 0.96)	ASH1 **M/G1 **(5, 0.94)
	AGA2 **M/G1 **(5, 0.88)	POL30 **G1 **(2, 0.97)	HTA3 **S **(6, 0.94)	EGT2 **M/G1 **(6, 0.94)
	TIP1 **M/G1 **(12, 0.85)	MNN1 **G1 **(3, 0.96)	CDC5 **M **(7, 0.94)	PCL9 **M/G1 **(8, 0.92)
	GFA1 **M/G1 **(22, 0.82)	CLN2 **G1 **(4, 0.96)	CLB2 **M **(11, 0.93)	SIC1 **M/G1 **(9, 0.91)
	GLK1 **M/G1 **(31, 0.81)	RNR1 **G1 **(5, 0.96)	BUD4 **M **(17, 0.93)	FAA3 **M/G1 **(14, 0.88)
		CDC21 **G1 **(10, 0.94)	KIP2 **G2 **(21, 0.92)	CDC6 **M/G1 **(20, 0.85)
		MCD1 **G1 **(11, 0.93)	ERG3 **G2 **(22, 0.92)	MCM3 **M/G1 **(24, 0.84)
		CTF4 **G1 **(12, 0.93)	CLB1 **M **(24, 0.92)	MCM2 **M **(25, 0.83)
		SMC3 **G1 **(13, 0.93)	ACE2 **M **(26, 0.92)	PHO11 **M **(26, 0.83)
		MSH2 **G1 **(15, 0.93)	MOB1 **M **(27, 0.91)	BUD9 **G1 **(27, 0.83)
		CDC9 **G1 **(16, 0.92)	AUR1 **G2 **(28, 0.91)	PHO12 **M **(33, 0.82)
		MSH6 **G1 **(18, 0.92)	ORC1 **G2 **(32, 0.91)	CDC47 **M **(34, 0.82)
		RFA2 **G1 **(19, 0.92)	TUB2 **G2 **(33, 0.91)	CDC46 **M/G1 **(35, 0.81)
		RAD27 **G1 **(20, 0.92)	SSO2 **G2 **(35, 0.90)	FTR1 **M **(36, 0.81)
		RAD51 **G1 **(21, 0.92)	CWP2 **G2 **(47, 0.90)	PHO5 **M **(38, 0.81)
		CSI2 **G1 **(24, 0.91)	NUM1 **G2 **(62, 0.89)	
		RNR3 **G1 **(25, 0.91)	MYO1 **M **(65, 0.89)	
		RAD53 **G1 **(26, 0.91)	NUF2 **M **(73, 0.89)	
		RSR1 **G1 **(29, 0.90)	TEM1 **M **(80, 0.89)	
		RFA1 **G1 **(30, 0.90)	CWP1 **G2 **(84, 0.89)	

N_tot _(N_match_) with r>0.8:	35 (5)	353 (92)	3395 (121)	43 (15)

Classification:	None	G1	S, G2, M	M/G1

Scores: G1	0.008	0.596	0.161	0.007
S	0.000	0.154	0.715	0.000
G2	0.000	0.000	0.696	0.000
M	0.000	0.000	0.539	0.081
M/G1	0.082	0.062	0.040	0.173

**Table 2:** Analysis of 5-component curve resolution results from Alpha-696 data. Cell-cycle related genes from Alpha-full that have a strong correlation (r>0.8) with extracted profiles are listed, along with the associated phase. See text for more details.

	Curve 1	Curve 2	Curve 3	Curve 4	Curve 5
	AGA2 **M/G1 **(3)	GLK1 **M/G1 **(6)	HHF2 **S **(1)	CLB2 **M **(2)	EGT2 **M/G1 **(2)
	AGA1 **M/G1 **(5)	CLB6 **G1 **(8)	HTA1 **S **(2)	CYK2 **M **(6)	BUD9 **G1 **(5)
	SST2 **M/G1 **(9)	MFA1 **G1 **(10)	HTB1 **S **(3)	CDC5 **M **(7)	SIC1 **M/G1 **(10)
	STE2 **M **(10)		HHF1 **S **(4)	IQG1 **M **(8)	FAA3 **M/G1 **(12)
	KAR4 **M/G1 **(13)		HHT1 **S **(5)	MYO1 **M **(13)	PCL9 **M/G1 **(13)
	FUS1 **M/G1 **(22)		HHT2 **S **(6)	SWI5 **M **(14)	ASH1 **M/G1 **(19)
	MFA2 **M **(24)		HTA2 **S **(7)	CLB1 **M **(15)	
			HTB2 **S **(9)	ALK1 **M **(16)	
			HHO1 **S **(11)	PHO3 **M **(17)	
			SPC98 **G1 **(13)	PHO5 **M **(18)	
			GAS1 **G1 **(14)	BUD4 **M **(19)	
			PSA1 **G1 **(17)	CDC20 **M **(20)	
			HTA3 **S **(18)	PMP1 **M **(28)	
			STU2 **S **(25)	PMA2 **M **(30)	
			WSC2 **S **(26)	MOB1 **M **(31)	
			GOG5 **S **(28)	PMA1 **M **(32)	
			SUR4 **G1 **(29)	PHO11 **M **(33)	
			RFA3 **G1 **(30)	CHS2 **M **(36)	
			PDS1 **G1 **(32)	CDC47 **M **(37)	
			PMT1 **G1 **(33)	ACE2 **M **(38)	

N_tot _(N_match_):	27 (7)	12 (3)	1398 (101)	80 (24)	20 (6)

Classification:	M/G1	G1	S	M	M/G1

Scores: G1	0.000	0.014	0.326	0.000	0.008
S	0.000	0.000	0.743	0.000	0.000
G2	0.000	0.000	0.443	0.025	0.000
M	0.028	0.000	0.080	0.331	0.000
M/G1	0.110	0.020	0.000	0.000	0.106

**Table 3:** Analysis of 4-component curve resolution results from Alpha-full data. Cell-cycle related genes from Alpha-full that have a strong correlation (r>0.8) with extracted profiles are listed, along with the associated phase. See text for more details.

	Curve 1	Curve 2	Curve 3	Curve 4
	AGA2 **M/G1 **(3, 0.93)	POL30 **G1 **(1, 0.96)	HTA3 **S **(3, 0.91)	PMA1 **M **(2, 0.95)
	AGA1 **M/G1 **(7, 0.87)	CLN2 **G1 **(2, 0.96)	HTA1 **S **(7, 0.90)	IQG1 **M **(6, 0.94)
	MFA1 **G1 **(8, 0.85)	MCD1 **G1 **(3, 0.95)	ERG3 **G2 **(8, 0.90)	FTR1 **M **(10, 0.93)
	STE2 **M **(10, 0.84)	RNR1 **G1 **(4, 0.95)	HTB1 **S **(9, 0.90)	CDC47 **M **(11, 0.93)
	SST2 **M/G1 **(14, 0.82)	SWI4 **M/G1 **(5, 0.94)	HHO1 **S **(12, 0.89)	VAP1 **M **(13, 0.93)
		RAD51 **G1 **(7, 0.93)	HHF2 **S **(14, 0.89)	MYO1 **M **(16, 0.92)
		CLB6 **G1 **(8, 0.93)	CWP2 **G2 **(24, 0.88)	PHO5 **M **(17, 0.92)
		RAD53 **G1 **(9, 0.93)	RFA3 **G1 **(27, 0.88)	MCM2 **M **(23, 0.92)
		SVS1 **G1 **(10, 0.93)	WSC2 **S **(28, 0.88)	PHO11 **M **(28, 0.92)
		CTF4 **G1 **(11, 0.93)	GDA1 **S **(29, 0.88)	PHO3 **M **(31, 0.92)
		MNN1 **G1 **(12, 0.92)	SUR4 **G1 **(35, 0.87)	CYK2 **M **(32, 0.92)
		MSH6 **G1 **(19, 0.91)	HHF1 **S **(36, 0.87)	FET3 **M **(33, 0.92)
		CDC9 **G1 **(21, 0.90)	HHT2 **S **(38, 0.87)	PMA2 **M **(36, 0.91)
		SMC3 **G1 **(22, 0.90)	HHT1 **S **(42, 0.87)	CLB1 **M **(40, 0.91)
		RFA1 **G1 **(25, 0.90)	TUB2 **G2 **(55, 0.87)	CDC20 **M **(47, 0.91)
		RAD27 **G1 **(26, 0.90)	AUR1 **G2 **(61, 0.86)	BUD4 **M **(64, 0.90)
		CLB5 **G1 **(29, 0.90)	MET17 **G2 **(67, 0.86)	SHE2 **M **(85, 0.90)
		RNR3 **G1 **(30, 0.90)	MET14 **S **(72, 0.86)	FAA3 **M/G1 **(98, 0.90)
		CDC21 **G1 **(31, 0.89)	KIP3 **G2 **(76, 0.86)	SWI5 **M **(121, 0.89)
		ASF1 **G1 **(33, 0.89)	GIC1 **S **(82, 0.86)	CDC6 **M/G1 **(130, 0.89)

N_tot _(N_match_) with r>0.8:	19 (5)	212 (60)	1517 (91)	3895 (125)

Classification:	M/G1	G1	S	M

Scores:				
G1	0.007	0.410	0.181	0.131
S	0.000	0.000	0.712	0.222
G2	0.000	0.000	0.591	0.516
M	0.014	0.000	0.136	0.758
M/G1	0.064	0.084	0.000	0.456
