# Supplementary material for: Multivariate curve resolution of time course microarray data
Source: BMC Bioinformatics. 2006 Jul 13;7:343. doi: 10.1186/1471-2105-7-343 (PMC1539028; doi:10.1186/1471-2105-7-343)
Supplement: Additional File 1 — Analysis of 7-component curve resolution results from Alpha-696 data. [file 1471-2105-7-343-S1.pdf]

Table A. Results of 7-component curve resolution analysis of Alpha-696 data showing significantly correlated genes from Spellman's classifications.

	Curve 1	Curve 2	Curve 3	Curve 4	Curve 5	Curve 6	Curve 7
	AGA1 **M/G1** (3)<br>AGA2 **M/G1** (7)<br>KAR4 **M/G1** (9)<br>FUS1 **M/G1** (16)<br>SST2 **M/G1** (19)<br>AFR1 **M/G1** (22)<br>STE2 **M** (23)		CLB6 **G1** (3)<br>GLK1 **M/G1** (7)	HHF1 **S** (1)<br>HTB1 **S** (2)<br>HHF2 **S** (3)<br>HTA1 **S** (4)<br>HHO1 **S** (5)<br>HHT2 **S** (6)<br>HHT1 **S** (8)<br>HTA2 **S** (11)<br>HTB2 **S** (16)<br>HTA3 **S** (21)<br>SPC98 **G1** (22)<br>WSC2 **S** (23)<br>YNK1 **G1** (24)<br>SUR4 **G1** (25)<br>RFA3 **G1** (28)<br>PMT1 **G1** (37)<br>GDA1 **S** (41)<br>PDS1 **G1** (45)	CLB2 **M** (1)<br>CDC5 **M** (5)<br>IQG1 **M** (8)<br>CYK2 **M** (9)<br>CLB1 **M** (12)<br>ALK1 **M** (13)<br>MYO1 **M** (14)<br>SWI5 **M** (16)<br>BUD4 **M** (17)<br>CDC20 **M** (19)<br>PHO3 **M** (23)<br>PMP1 **M** (24)<br>MOB1 **M** (25)<br>CHS2 **M** (26)<br>PMA2 **M** (29)<br>ACE2 **M** (31<br>PHO5 **M** (33)<br>PMA1 **M** (34)<br>KIP2 **G2** (36)<br>CDC47 **M** (39)	EGT2 **M/G1** (3)<br>ASH1 **M/G1** (6)<br>PCL9 **M/G1** (7)<br>SIC1 **M/G1** (11)	CTS1 **G1** (2)<br>BUD9 **G1** (7)<br>MSB2 **G1** (9)<br>PSA1 **G1** (10)
N_{tot} (N_{match})	23 (7)	7 (0)	8 (2)	45 (18)	89 (29)	14 (4)	12 (4)
Classification:	M/G1	None	G1	S	M	M/G1	G1
Scores:							
G1	0.000	0.000	0.007	0.041	0.000	0.000	0.028
S	0.000	0.000	0.000	0.281	0.000	0.000	0.000
G2	0.000	0.000	0.000	0.000	0.144	0.000	0.000
M	0.013	0.000	0.000	0.000	0.331	0.000	0.000
M/G1	0.130	0.000	0.020	0.000	0.000	0.087	0.000
