# Supplementary material for: Multivariate curve resolution of time course microarray data
Source: BMC Bioinformatics. 2006 Jul 13;7:343. doi: 10.1186/1471-2105-7-343 (PMC1539028; doi:10.1186/1471-2105-7-343)
Supplement: Additional File 2 — Analysis of 5-component curve resolution results from Alpha-full data. [file 1471-2105-7-343-S2.pdf]

Table B. Results of 5-component curve resolution analysis of Alpha-Full data showing significantly correlated genes from Spellman's classifications.

	Curve 1	Curve 2	Curve 3	Curve 4	Curve 5
	AGA2 **M/G1** (4, 0.94)	POL30 **G1** (1, 0.95)	HTA3 **S** (4, 0.94)	MET6 **S** (8, 0.88)	ASH1 **M/G1** (8, 0.91)
	AGA1 **M/G1** (5, 0.90)	CLN2 **G1** (2, 0.95)	ERG3 **G2** (6, 0.92)	SSO2 **G2** (33, 0.87)	SIC1 **M/G1** (9, 0.91)
	MFA1 **G1** (9, 0.84)	MCD1 **G1** (3, 0.94)	ALK1 **M** (7, 0.92)	HST3 **M** (79, 0.86)	PCL9 **M/G1** (10, 0.91)
	STE2 **M** (11, 0.82)	RNR1 **G1** (4, 0.93)	AUR1 **G2** (19, 0.90)	KIP2 **G2** (290, 0.85)	FAA3 **M/G1** (12, 0.90)
	SST2 **M/G1** (13, 0.81)	SWI4 **M/G1** (6, 0.93)	CDC5 **M** (24, 0.90)	SHE2 **M** (307, 0.85)	FTR1 **M** (18, 0.88)
		CLB6 **G1** (7, 0.92)	TUB2 **G2** (30, 0.89)	BUD4 **M** (308, 0.84)	EGT2 **M/G1** (20, 0.88)
		RAD53 **G1** (8, 0.92)	KIP2 **G2** (33, 0.89)	CHS6 **G2** (410, 0.84)	MCM2 **M** (21, 0.87)
		RAD51 **G1** (9, 0.92)	MOB1 **M** (36, 0.89)	PMI40 **S** (454, 0.84)	CDC47 **M** (22, 0.87)
		SVS1 **G1** (10, 0.92)	ORC1 **G2** (37, 0.89)	PMP1 **M** (478, 0.84)	PHO11 **M** (24, 0.86)
		CTF4 **G1** (11, 0.91)	CWP2 **G2** (43, 0.89)	APC1 **M** (531, 0.83)	FET3 **M** (25, 0.86)
		MNN1 **G1** (15, 0.90)	HHO1 **S** (45, 0.88)	NUF2 **M** (559, 0.83)	PHO5 **M** (28, 0.86)
		RNR3 **G1** (17, 0.89)	CLB2 **M** (48, 0.88)	WHI3 **G2** (632, 0.83)	PMA1 **M** (29, 0.86)
		SMC3 **G1** (21, 0.89)	GDA1 **S** (49, 0.88)	ACE2 **M** (652, 0.83)	VAP1 **M** (38, 0.85)
		CDC9 **G1** (22, 0.89)	ACE2 **M** (57, 0.88)	BUD3 **G2** (683, 0.83)	FAA4 **M/G1** (40, 0.85)
		CSI2 **G1** (23, 0.89)	BUD4 **M** (62, 0.88)	SUC2 **M** (699, 0.83)	BUD9 **G1** (43, 0.85)
		MSH6 **G1** (24, 0.89)	ALG7 **G2** (70, 0.88)	ALK1 **M** (704, 0.83)	MCM3 **M/G1** (44, 0.85)
		RFA1 **G1** (25, 0.89)	KIP3 **G2** (74, 0.88)	PMA1 **M** (743, 0.83)	PHO12 **M** (45, 0.85)
		CDC21 **G1** (29, 0.88)	TEM1 **M** (77, 0.88)	SAS3 **G2** (790, 0.83)	CDC6 **M/G1** (47, 0.85)
		MSH2 **G1** (30, 0.88)	SSO2 **G2** (79, 0.88)	SWI5 **M** (844, 0.82)	PHO3 **M** (62, 0.83)
		CLB5 **G1** (32, 0.88)	MET17 **G2** (82, 0.88)	SUT1 M (855, 0.82)	IQG1 **M** (63, 0.83)
N_{tot} (N_{match})	13 (5)	155 (53)	2445 (107)	2046 (46)	271 (31)
Classification:	M/G1	G1	S, G2, M	G2, M	M, M/G1
Scores:					
G1	0.007	0.356	0.154	0.027	0.021
S	0.000	0.000	0.716	0.178	0.000
G2	0.000	0.000	0.637	0.315	0.000
M	0.013	0.000	0.389	0.270	0.208
M/G1	0.064	0.083	0.020	0.020	0.272
